# Supplementary material for: WAT-on-a-chip integrating human mature white adipocytes for mechanistic research and pharmaceutical applications
Source: Sci Rep. 2020 Apr 20;10:6666. doi: 10.1038/s41598-020-63710-4 (PMC7170869; doi:10.1038/s41598-020-63710-4)
Supplement: Supplementary file 1 — Supplementary information. [file 41598_2020_63710_MOESM1_ESM.docx]

Supplementary information:

WAT-on-a-chip integrating human mature white adipocytes for mechanistic research and pharmaceutical applications

Julia Rogal^a,b^, Carina Binder^a^, Elena Kromidas^a^, Julia Roosz^a^, Christopher Probst^a^, Stefan Schneider^a^, Katja Schenke-Layland^b,c,d^, Peter Loskill^a,b #^

^a^ Fraunhofer Institute for Interfacial Engineering and Biotechnology IGB, Nobelstrasse 12, 70569 Stuttgart, Germany

^b^ Department of Women’s Health, Research Institute for Women’s Health, Eberhard Karls University, Calwerstrasse 7, 72076 Tübingen, Germany

^c^ NMI Natural and Medical Sciences Institute at the University of Tübingen, Markwiesenstr. 55, 72770 Reutlingen, Germany

^d^ Department of Medicine/Cardiology, Cardiovascular Research Laboratories, David Geffen School of Medicine at UCLA, 675 Charles E. Young Drive South, MRL 3645, Los Angeles, CA, USA

# Correspondence to:

Jun. Prof. Dr. Peter Loskill

Fraunhofer Institute for Interfacial Engineering and Biotechnology IGB

Nobelstrasse 12, 70569 Stuttgart, Germany

**Microfabrication of the microfluidic platform**

**Replica molding of PDMS parts**

Polydimethylsiloxane (PDMS; Sylgard 184, Dow Corning, USA) layers were created *via* replica molding (cf. **figure S1**) of a 10:1 (w/w) mixture of PDMS pre-polymer and curing agent. To create the PDMS layer containing the media channel structures by standard molding, 25 g of the uncured PDMS mix was poured on top of the wafer and stored overnight at 60 °C for curing of the PDMS. On the next day, after peeling off the standard-molded slab of PDMS from the media wafer, inlets and outlets were created using a biopsy punch (diameter: 0.75 mm; World Precision Instruments). In contrast, an exclusion molding technique was used to create the PDMS layer incorporating the open tissue-chamber structures: ~ 1 g of the uncured PDMS/curing agent mixture was poured on top of the micropatterned wafer and spread by gently tilting and rotating the wafer. A pre-cut, rounded piece of 3M Scotchpak™ 1022 Release Liner Fluoropolymer Coated Polyester Film (3M ID 70000200280, St. Paul, MN, USA) was lowered onto the PDMS mixture, with its treated surface face-down. The ensemble was clamped between to smooth surfaces (e.g. unpatterned wafers), and stored overnight at 60 °C for curing of the PDMS. The exclusion-molded PDMS structures were carefully examined, and voided of residues of PDMS adhering to the foil, that might close off the open structures.


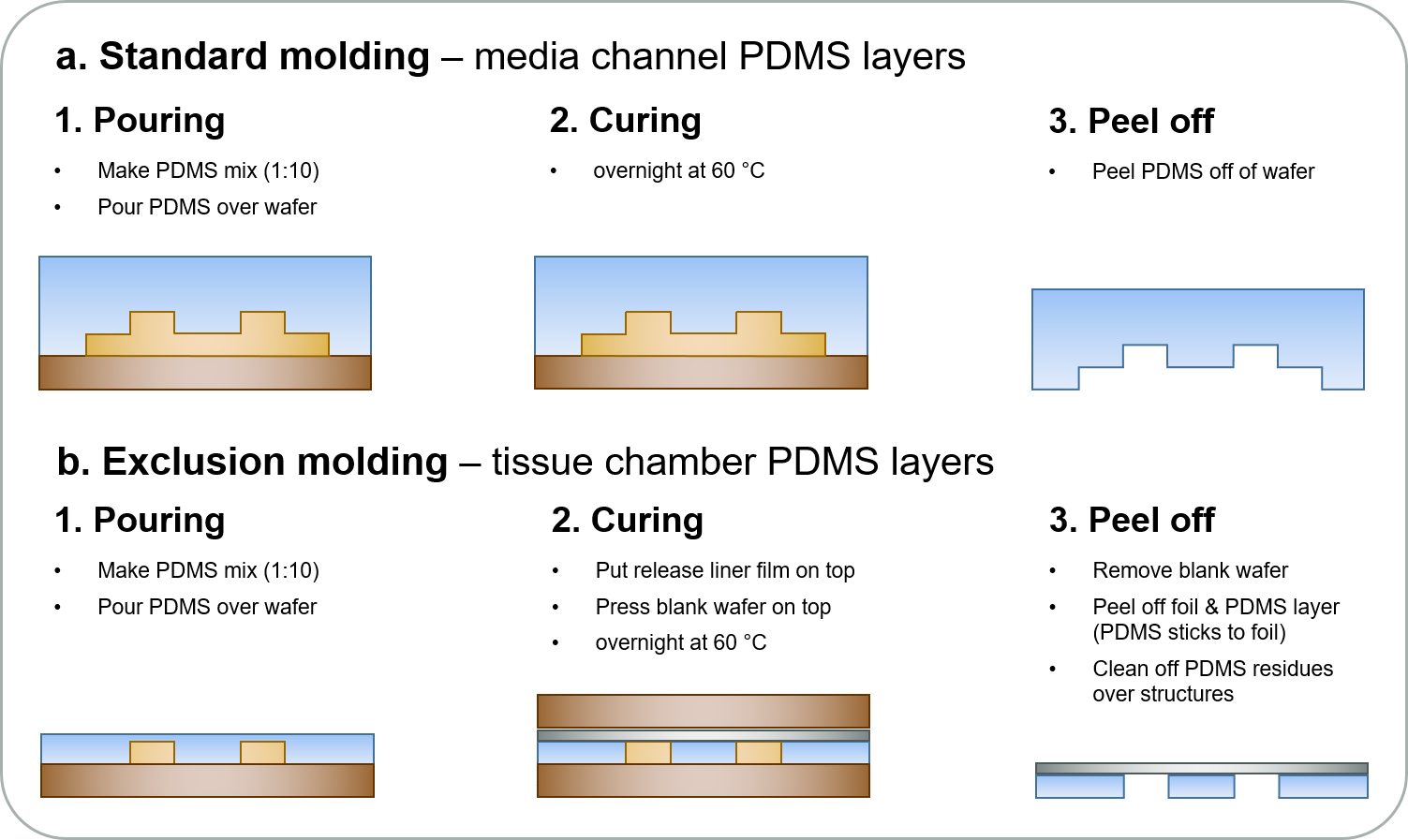


**Fig S1: Molding techniques for fabricating the WAT-chip. a.** PDMS slabs featuring media channel microstructures were produced by a standard molding process. First, 25 g of a PDMS mixture (containing a 10:1 w/w ratio of PDMS pre-polymer and curing agent) was poured over the media wafer. Then, after curing overnight at 60 °C, the PDMS mold was peeled off the wafer. **b.** PDMS layers featuring the tissue chamber systems were fabricated by exclusion molding. For this procedure, only ~ 1 g of the PDMS mixture was poured over the microstructured wafer. Then, a release liner, consisting of a polyester film, coated with fluoropolymer, was placed on top of the PDMS mixture, with the coated side facing the PDMS. A blank wafer was pressed against the other side of the foil to create open PDMS structures. After peel-off, PDMS stuck to the foil and PDMS residues were gently removed to uncover the intended open structures.

**Membrane functionalization**

Commercially available PET-membranes (TRAKETCH® PET 3.0 p S210x300, SABEU GmbH & Co. KG, Northeim, Germany) were modified using a plasma-enhanced chemical vapor deposition (PECVD) process. The PECVD, carried out by a low-pressure plasma, was performed in a custom-built plate reactor. The plasma was generated using a high-frequency (HF) source at 13,56 MHz (CESAR™ Generator Model 136, Dressler Hochfrequenztechnik GmbH, Stolberg, Germany) and a matching box (AE VarioMatch™ Match Network, VM 1500 Platform, Dressler Hochfrequenztechnik GmbH, Stolberg, Germany). The PECVD reactor was evacuated by a rotary vane vacuum pump (TRIVAC D 40B, Leybold GmbH, Köln, Germany) and a roots vacuum pump (RUVAC WS 251, Leybold GmbH, Köln, Germany). Pressure measurements were carried out using a vacuum gauge controller (Granville-Phillips® Series 375 Convectron®, MKS Instruments, Andover, MA, United States). All gas flows into the reactor were controlled by mass-flow controllers (Type 1259, MKS Instruments, Andover, MA, United States) and a multi-gas controller (Type 647B, MKS Instruments, Andover, MA, United States). The precursor Hexamethyldisiloxane (HMDSO) was evaporated at 37 °C and led into the reactor controlled by a mass-flow controller (UFC-7300, UNIT Instruments Inc., Austin, TX, United States). Before initiation of the coating process, the reactor chamber was cleaned using isopropanol, and all gas lines were evacuated to remove any residual gas. The PET-membranes, sized 210 × 300 mm², were placed inside the reactor chamber on top of a piece of protection paper of the same size, provided by the supplier. The plasma reactor was closed and evacuated until a pressure of approximately 1 - 2 µbar was reached. Before starting the PECVD, the membranes were pre-treated by a 30 s exposure to an oxygen plasma to clean and activate the substrate. The process parameters are listed in table S1. Immediately after the pre-treatment, the membranes were coated by PECVD for 40 s, using the parameters in table S1. The pre-treatment and coating process were applied on both sides of the membranes. After the membrane functionalization, the membranes were cut to the desired size, using a CO_2_-laser-cutter (VLS2.30, Universal Laser Systems).

**Table S1** PECVD process parameters

| **Process** | **Time [s]** | **Power [W]** | **Pressure [µbar]** | **Gas flows [sccm]** | | |
| --- | --- | --- | --- | --- | --- | --- |
|  |  |  |  | **O_2_** | **N_2_** | **HMDSO** |
| Pre-treatment | 30 | 70 | 63 | 100 | 0 | 0 |
| PECVD | 40 | 140 | 87 | 0 | 100 | 7 |

*Adhesion test of PET to PDMS bonding*

Peel tests were carried out to assess the bonding strength between the functionalized membranes and the PDMS. Membranes were lasercut in pieces sized 115 × 32 mm². After washing with 70 % ethanol, drying and fixing to a 175 µm thick PET foil, membranes and PDMS were plasma activated and bonded, as described above. After the completion of bonding, 180° peel tests (width B = 32 mm) were conducted using a tensile testing machine (Z005; Zwick Roell, Ulm, Germany) and a 2,5 kN nominal force load cell (XForce HP, Zwick Roell, Ulm, Germany). To test the longtime stability of bonding, membranes bonded to PDMS were kept in PBS- for 7 days at room temperature.

The membranes stored for 7 days in PBS^-^ and membranes tested straight after bonding were compared (figure S2) and no weakening of the bonding was observed. In contrast to conventional membrane bonding approaches, this PECVD-based bonding technique allows for a facile generation of PDMS-PET hybrids that is easily scalable.


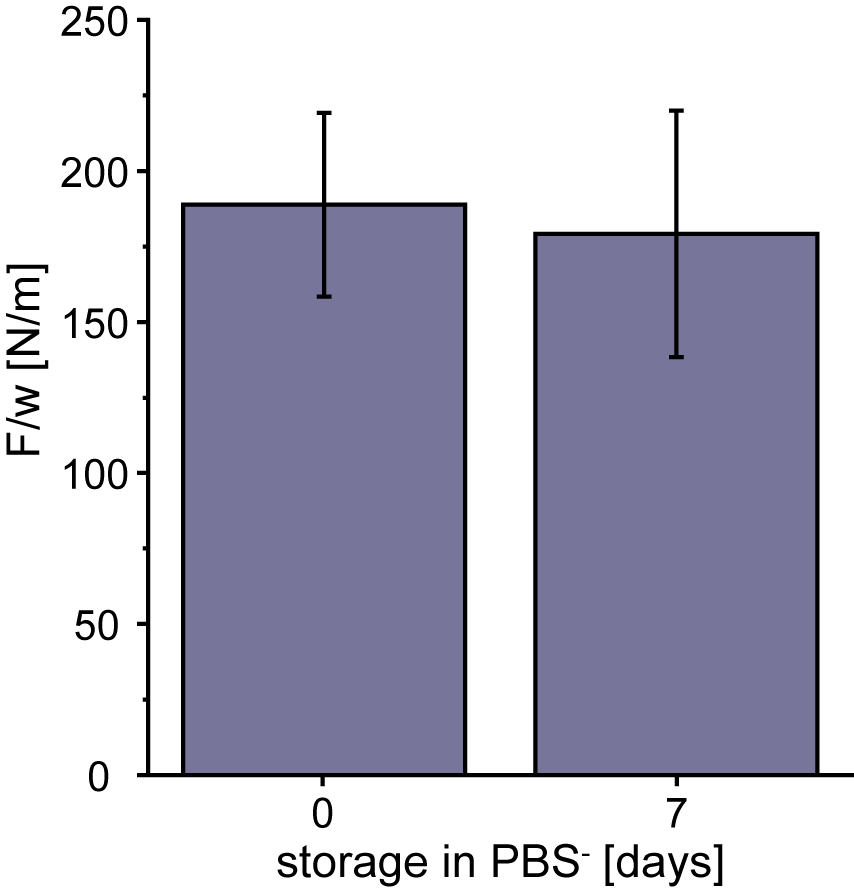


**Fig S2: Adhesion test of PET to PDMS bonding.** As confirmed by 180° peel tests, the bonding strength between PECVD-funtionalized PET membranes and PDMS was not compromised by long-term storage in PBS^-^.

**Chip assembly**

Prior to chip assembly, all PDMS parts were thoroughly rinsed with isopropanol, blow-dried with N_2_, and further cleaned by repeated pressing and peeling with strips of household adhesive tape. In a three-step process (cf. figure S3) of O_2_-plasma activation (15 s, 50 W; Diener Zepto, Diener electronic GmbH + Co. KG, Ebhausen, Germany) and bonding, the exclusion-molded layer was bonded to a microscope slide (bonding enhancement for 30 min at 60°C) (1), the functionalized PET-membrane was bonded into the insert within the lop layer (2), and finally, the top layer and membrane were bonded to the bottom layer on the glass slide (3). Alignment of structures in top and bottom layer was conducted under visual guidance of a stereomicroscope. To enhance bonding, the chip was firmly pressed together between two microscope slides using foldback clips and kept overnight in an oven at 60°C. To verify the sealing tightness and fluidic connectivity of the system, tissue chambers and media channels were perfused with 70% ethanol.


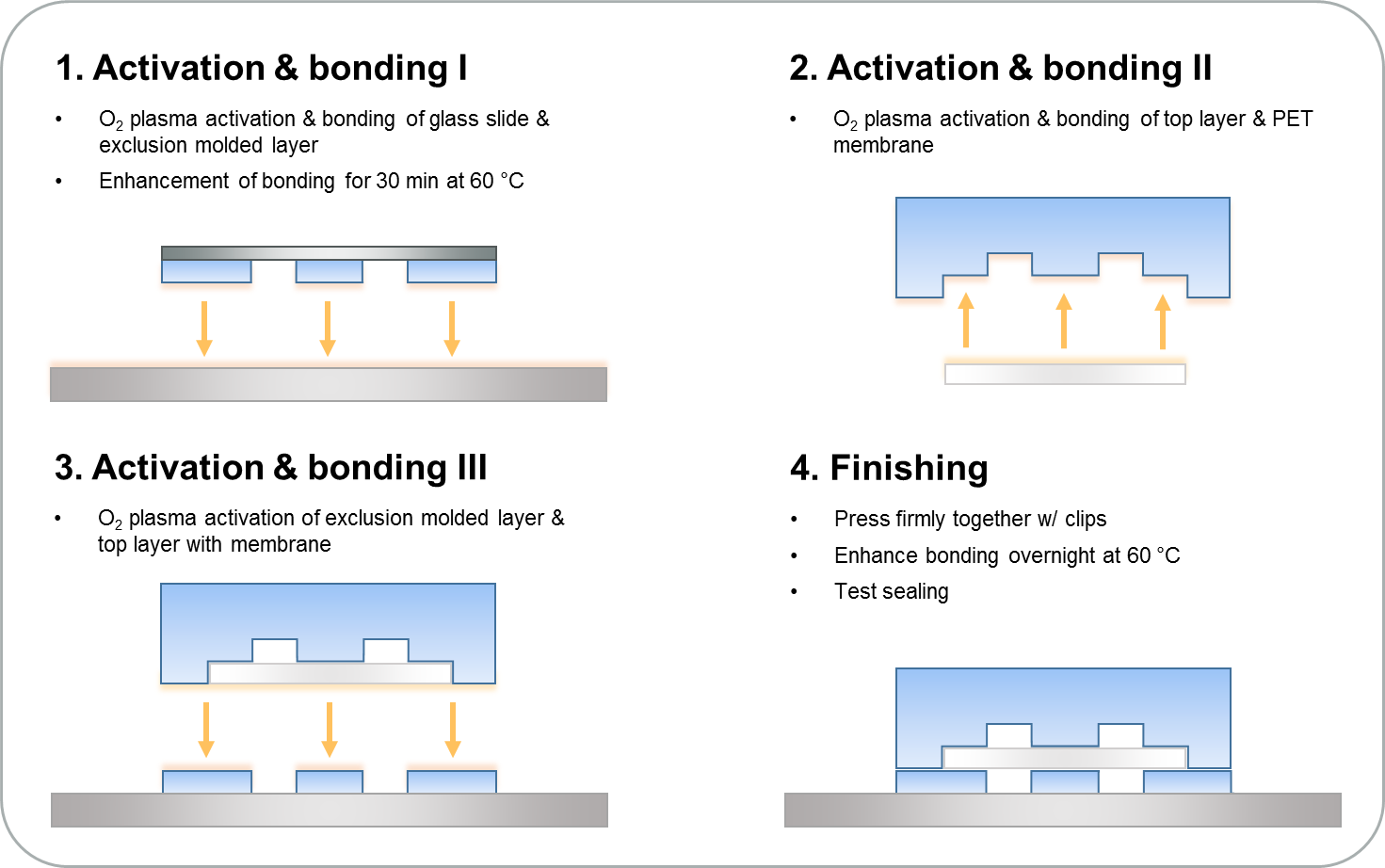


**Fig S3: Chip assembly supported by plasma activation.** The WAT-chip was assembled in three subsequent O_2_-plasma activation (15 s, 50 W) and bonding steps: 1. Bonding of the exclusion-molded layer to a microscope slide and enhancement of bonding for 30 min at 60 °C. 2. Bonding of the PET membrane to the standard-molded PDMS layer. 3. Bonding of the top layer to the exclusion-molded layer. 4. Overnight enhancement of bonding at 60 °C. To test sealing strength, and fluidic continuity, 70% ethanol was flushed through the systems.

**Influence of human serum albumin presence on non-esterified fatty acid analysis**

To ensure that increases in the measured NEFA concentrations could not be attributed to the presence of HSA during assay performance, we performed NEFA-measurements of the oleic acid standard with and without HSA supplemented medium. Oleic acid standard (100 µM) was added to AM-1 only or to AM-1 supplemented with 0.2% (w/v) HSA, and the NEFA assay was performed as described. No differences were observed in the measured absorbances between the two conditions (**figure S4**).


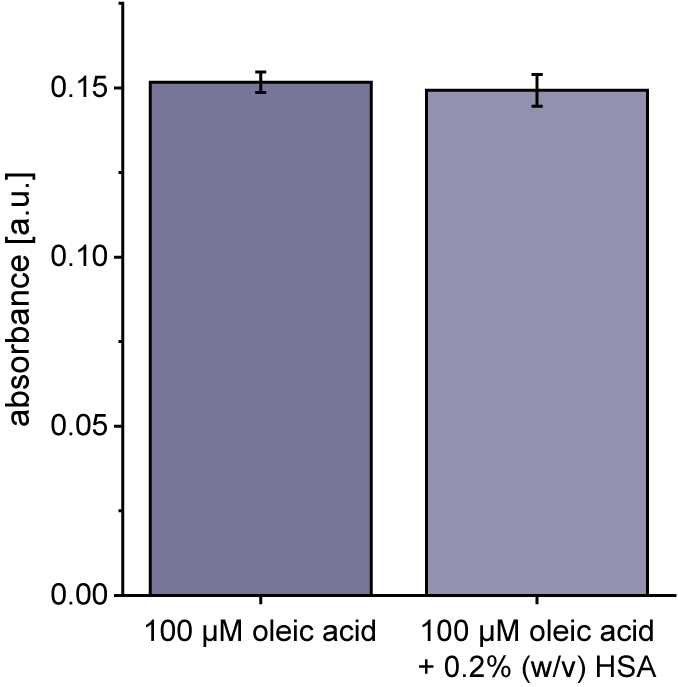


**Fig S4: Influence of HSA presence on NEFA measurement.** The presence of 0.2% (w/v) HSA in the culture medium did not influence the enzymatic analysis of the NEFAs.

**Absorption of adipokines into PDMS**

To characterize the extent of adipokine absorption into the PDMS, we determined the change in adiponectin levels after perfusion through the WAT-chip. A 3125 pg/ml adiponectin solution was injected into the PDMS platform (chambers filled with hydrogel, but no cells) and perfused for 16 h. After 16 h, the adiponectin concentration was determined using LEGENDplex™ Human Metabolic Panel 1 Kit (Biolegend^®^, San Diego, CA, United States) according to the manufacturer’s instructions. Perfusion through the chip decreased the adiponectin concentration considerably revealing a strong absorption of adiponectin by the PDMS.


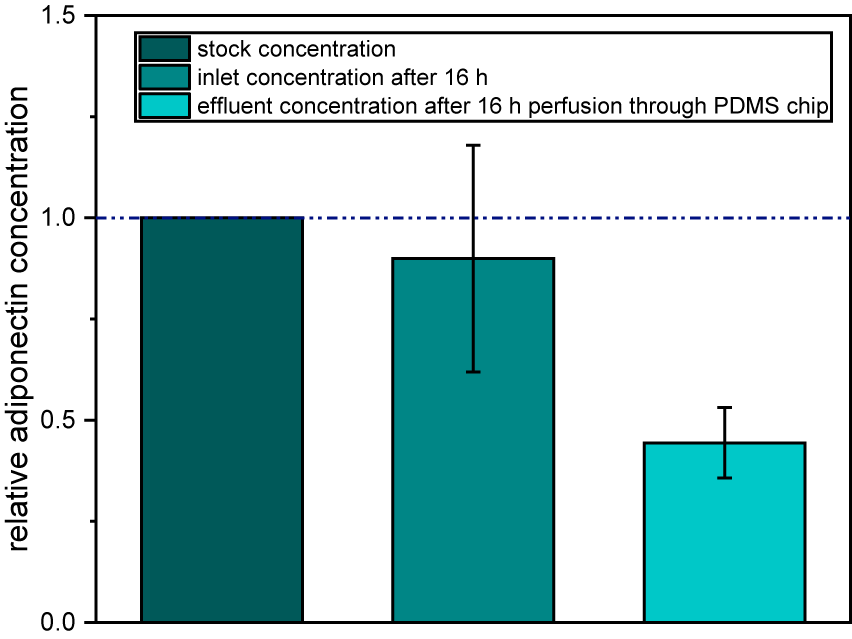


**Fig S5: Impact of the PDMS chip on adipokine detection.** An adiponectin stock solution (3125 pg/ml) was perfused through the microfluidic platform (tissue chambers filled with collagen 1 hydrogel, but no cells) for 16 h. After 16 h, the adiponectin concentration measured from the chips’ effluents was considerably decreased compared to inlet- and stock concentration (n = 2 systems with 8 chambers each).
